# The influence of substance abuse on inhibition capacities and risky decision in a group of outpatient schizophrenia patients

**DOI:** 10.1192/j.eurpsy.2023.2297

**Published:** 2023-07-19

**Authors:** P. Dannon, S. Kertzman

**Affiliations:** Psychiarty, Herzog Hospital, Tel Aviv, Israel

## Abstract

**Introduction:**

Substance abuse is common among patients with schizophrenia, is related toworse course and outcome of illness. Unfortunately, little is known about how substanceabuse affects the cognitive function of schizophrenia patients, whose cognitive function isoften already comprised. Neurocognitive functioning includes inhibition control and decision-making, and both schizophrenia and substance use disorder are related to impairmentsof inhibition control. However, the influence of substance abuse on inhibition capacitiesamong schizophrenia patients is unclear

**Objectives:**

This study measured the influence of substance use disorder on inhibition capacities and risky decision-making in a group of 39 schizophrenia patients that were evaluated using a socio-demographic questionnaire and clinical assessment using the Positive and Negative Syndromes Scale for Schizophrenia. To assess inhibition control we utilized the Matching Familiar Figure Test (MFFT) and the Stroop task, and to evaluate decision-making we used the Iowa Gambling Task (IGT) and self-report questionnaire, the Barratt Impulsiveness Scale

**Methods:**

Univariate analysis found significant differences between the groups with regard to criminal history (v2¼5.97, p¼.015), smoking status (v:12.30, p<.001), and total BIS score (t: 2.69, df¼37, p¼.01). Our model did not find a significant effect of substance abuse on the first response time and number of errors on the MFFT or in the total interference index of Stroop performance and net score on risky decision-making in the IGT.

**Results:**

The two groups did not differ significantly either in first response time or in number of errors on the MFFT (F¼0.54, p¼.47, d¼0.24, 95% CI [-0.4, 0.88]; F¼0.28, p¼.60, d¼0.61, 95% CI [0, 1.26], respectively), nor did they differ in the total interference index of the Stroop task (F(1)¼0.49, p¼.49, d¼0.25, 95% CI [-0.38, 0.88]).

**Conclusions:**

The analyses did not detect any statistically significant effect of substance abuse on inhibition control or risky decision-making processes in outpatients diagnosed with schizophrenia, despite increased impulsivity, criminal history and smoking status. These results neither support nor disprove previous findings.

**Disclosure of Interest:**

None Declared

